# Supplementary material for: CRIF1-CDK2 Interface Inhibitors Enhance Taxol Inhibition of the Lethal Triple-Negative Breast Cancer
Source: Cancers (Basel). 2022 Feb 16;14(4):989. doi: 10.3390/cancers14040989 (PMC8870401; doi:10.3390/cancers14040989)
Supplement: Supplementary file 1 [file cancers-14-00989-s001.zip › cancers-1558749-supplementary.pdf]

Supplementary materials

CRIF1–CDK2 Interface Inhibitors Enhance Taxol Inhibition of the Lethal Triple-Negative Breast Cancer

Xiaoye Sang, Nassira Belmessabih, Ruixuan Wang, Preyesh Stephen and Sheng-Xiang Lin

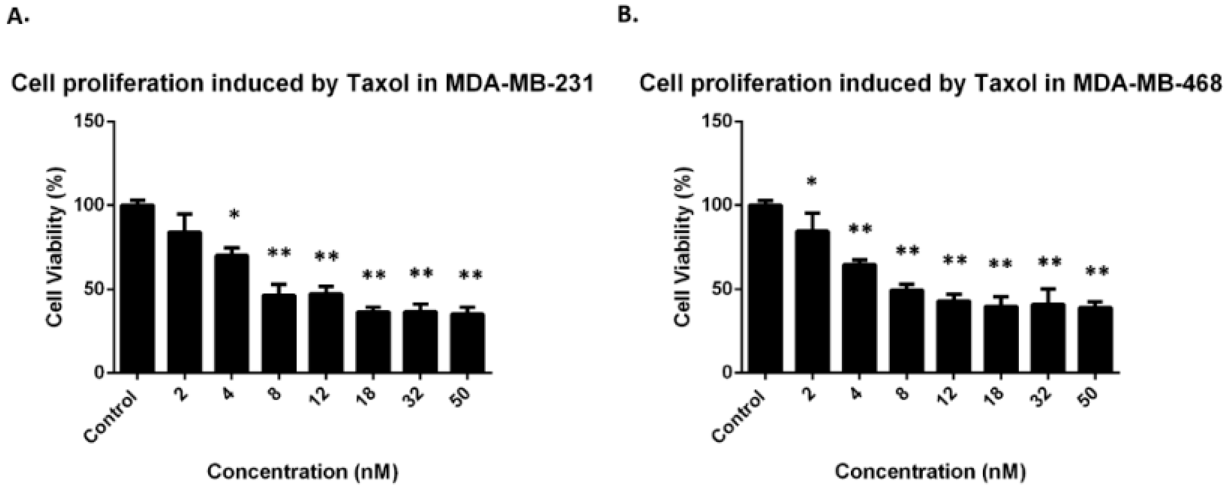

Figure S1. Cell proliferation in response to Taxol in TNBC cell lines. Cell proliferation was determined by Cell Proliferation Reagent WST-1 in MDA-MB-231 (**A**) and MDA-MB-468 (**B**) cells treated with different concentrations of Taxol for 4 days. Data are reported as % of DNA synthesis vs. control (100%). Each point represents the mean of four replications (mean ± SD). Statistical significance by one-way ANOVA: * $p < 0.05$ vs. control (Ctrl); ** $p < 0.01$ vs. control (Ctrl).

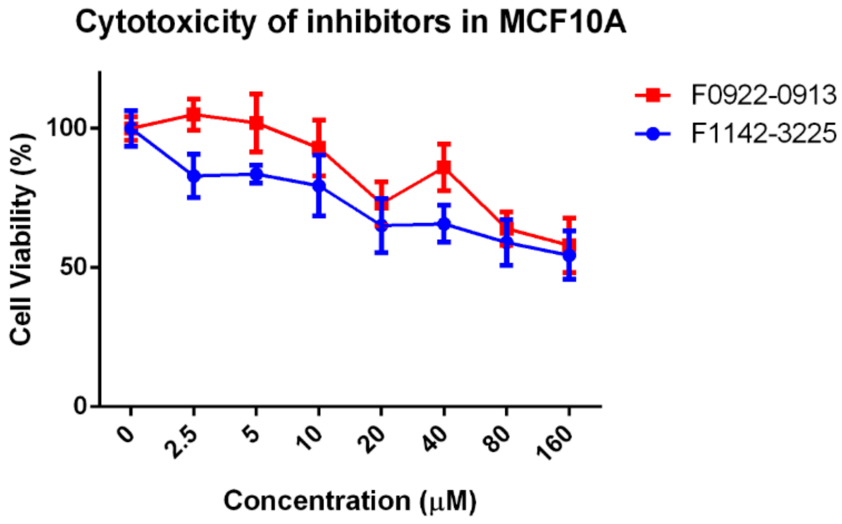

Figure S2. Cytotoxicity of inhibitors in MCF10A. The cytotoxicity of F1142-3225 and F0922-0913 in MCF10A cells were evaluated by Cell Proliferation Reagent WST-1. Cells were treated with inhibitors for 4 days. Data are reported as % of DNA synthesis vs. control (100%). Each point represents the mean of four replications (mean ± SD).

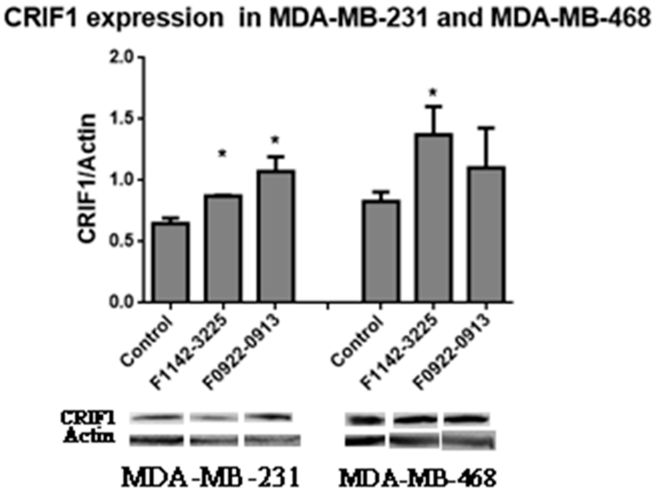

Figure S3. The CRIF1 protein expression in TNBC cell lines. CRIF1 protein expression after treatment with F1142-3225 or F0922-0913 for 48 h was determined by western blot in MDA-MB-231 cells and MDA-MB-468 cells. Data are reported as the mean ± SD ($n = 3$) of the individual experiments. Statistical significance was determined by Student's test: * $p < 0.05$ vs. control (Ctrl).

MDA-MB-231

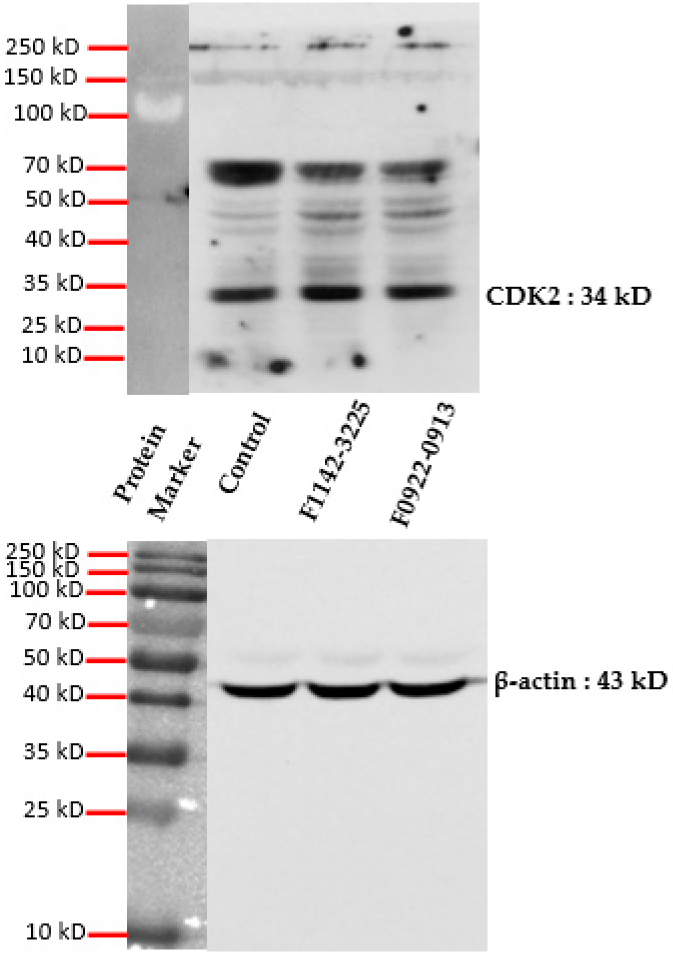

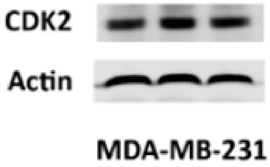

Measurements	CDK2	Actin	CDK2/Actin
**Control**	984	2709	0.363
**F1142-3225**	1432	2632	0.544
**F0922-0913**	1196	2352	0.508

MDA-MB-468

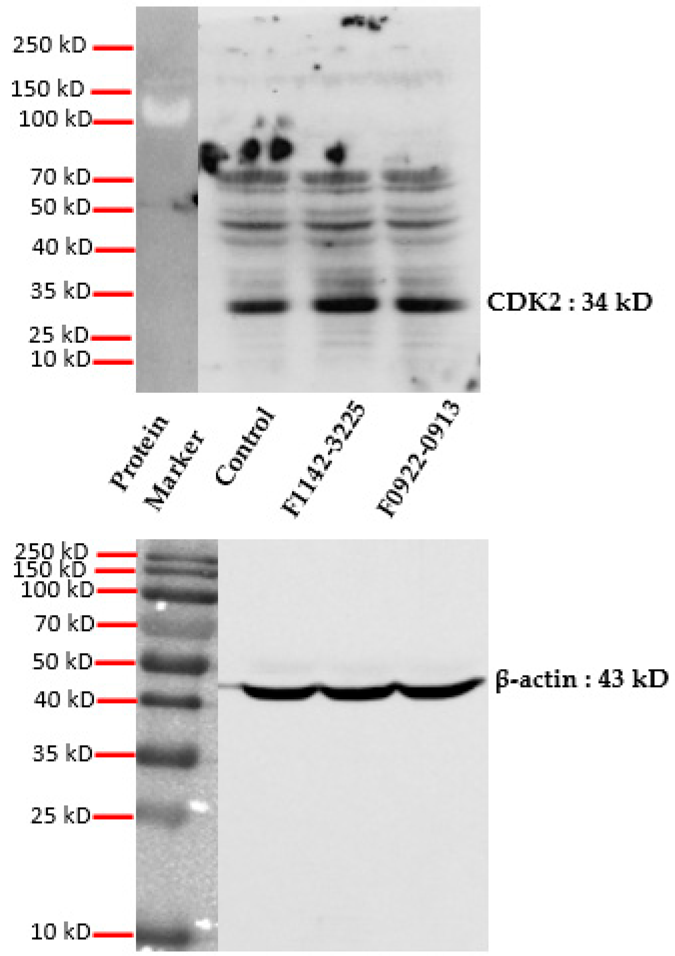

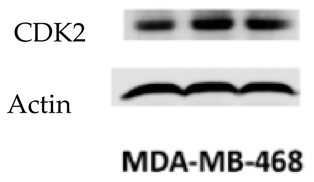

Measurements	CDK2	Actin	CDK2/Actin
**Control**	991	3232	0.306
**F1142-3225**	1637	3487	0.469
**F0922-0913**	1372	3798	0.361

Whole blot for **Figure 3B**. Whole blot membrane showing bands with all molecular weight markers.

MDA-MB-231

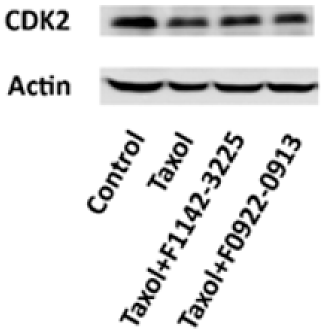

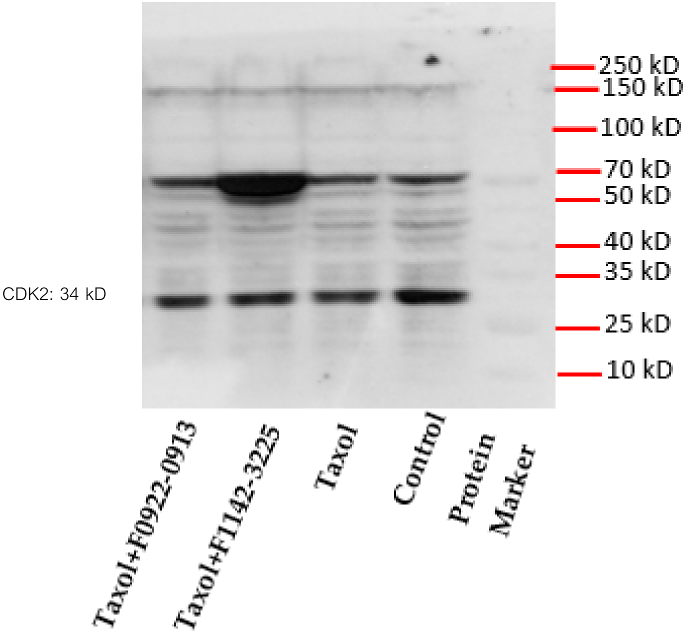

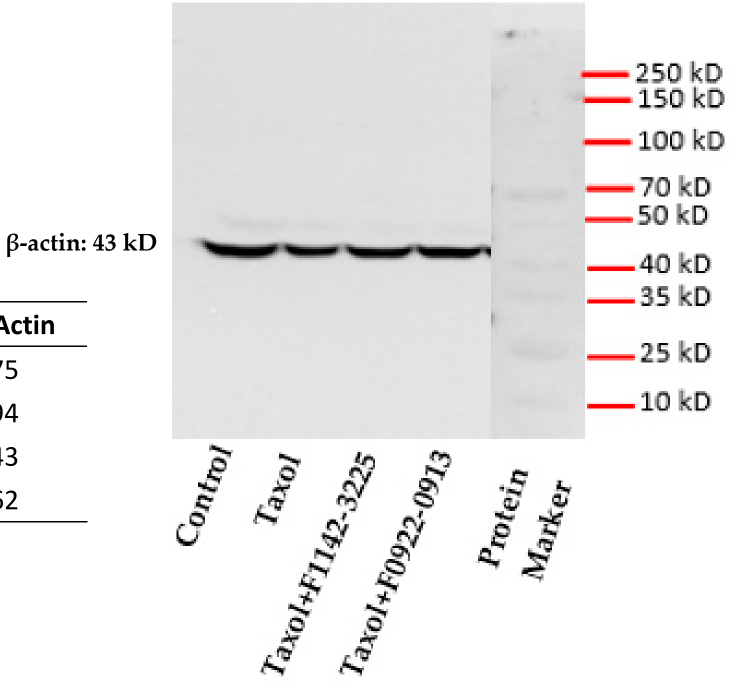

Measurements	CDK2	Actin	CDK2/Actin
**Control**	1668	2896	0.575
**Taxol**	816	1372	0.594
**Taxol+3225**	998	2907	0.343
**Taxol+0913**	896	2477	0.362

Whole blot for **Figure 7B**. Whole blot membrane showing bands with all molecular weight markers.

MCF-10A

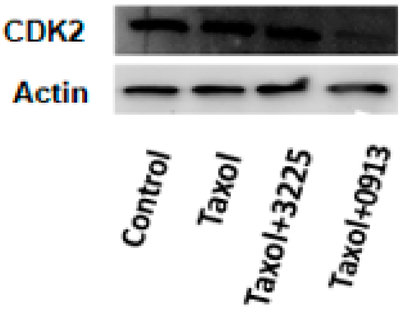

Measurements	CDK2	β-actin	CDK2/Actin
**Control**	44550.62	165248.38	0.269
**Taxol**	39866.84	157631.88	0.253
**Taxol+3225**	39740.85	157800.88	0.252
**Taxol+0913**	38015.14	162796.31	0.233

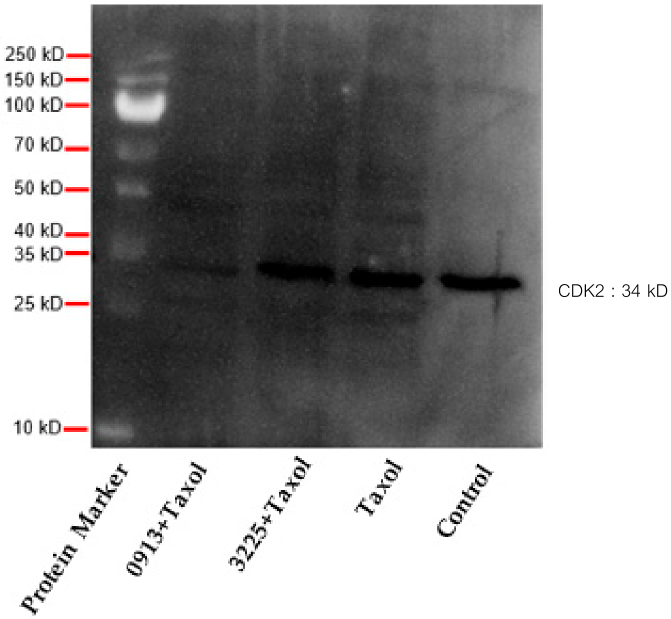

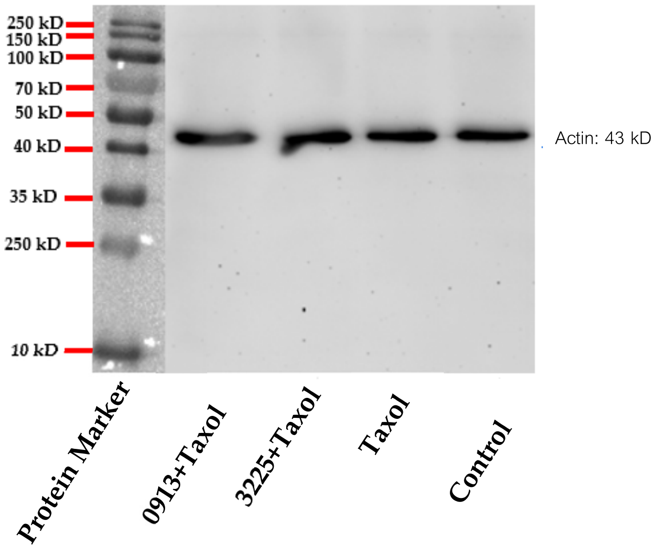

Whole blot for **Figure 7C.** Whole blot membrane showing bands with all molecular weight markers.

MDA-MB-231

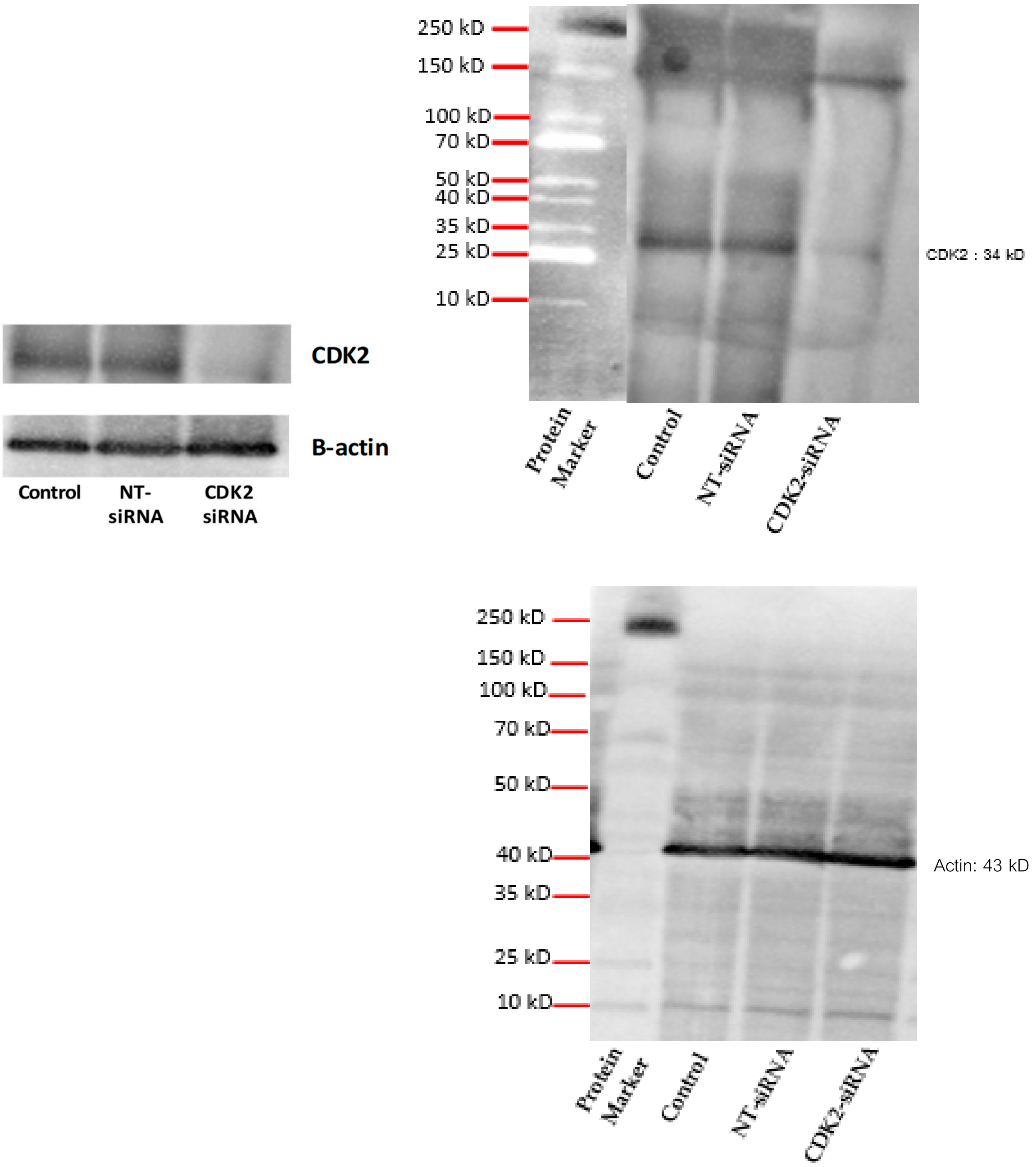

Whole blot for **Figure 8C.** Whole blot membrane showing bands with all molecular weight markers.

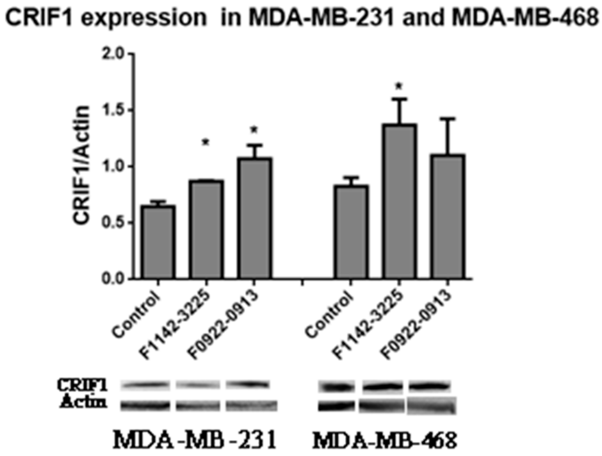

MDA-MB-231

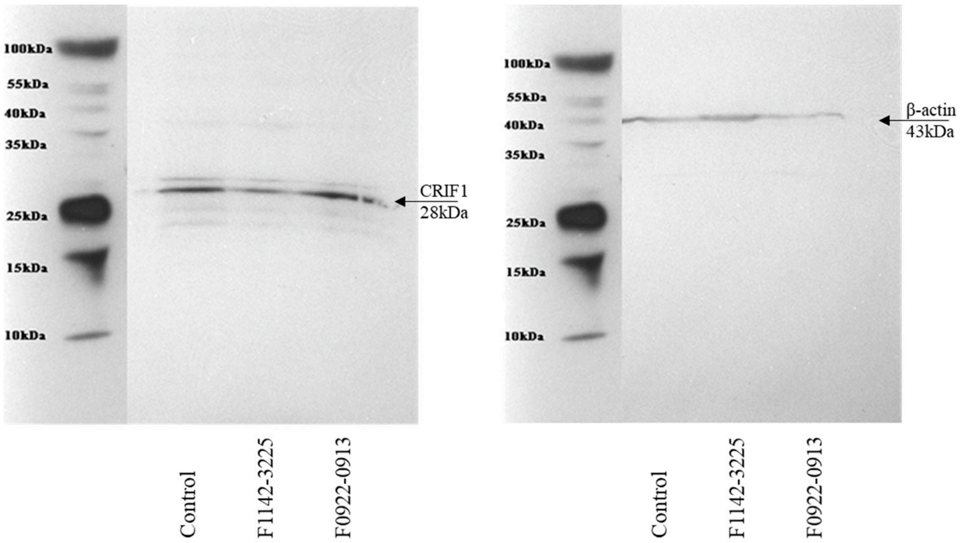

MDA-MB-468

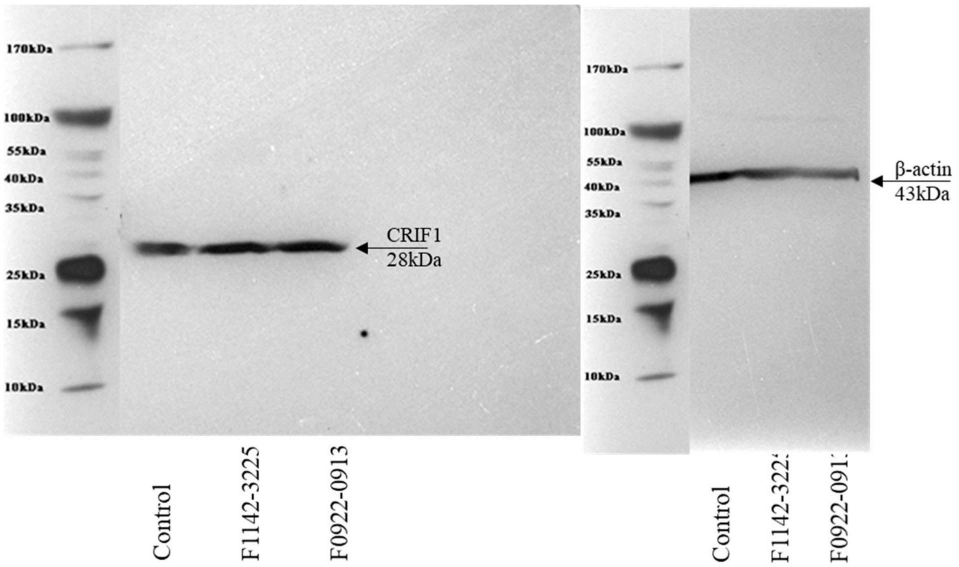

Whole blot for **Figure S3.** Whole blot membrane showing bands with all molecular weight markers.

Densitometry reading and density ratio:

Measurements	Inhibitor or Control	CRIF1	B-Actin	CRIF1/β-Actin (Mean Value for Repeat Experiments)
MDA-MB-231	Control	256,000	414,000	0.649796
	F1142-3225	164,000	187,000	0.874332
	F0922-0913	246,000	249,000	1.072579
MDA-MB-468	Control	553,000	613,000	0.826922
	F1142-3225	670,000	485,000	1.372412
	F0922-0913	566,000	433,000	1.104548

Table S1. Inhibitor characteristics.

Compound	Protein Target	Chemical Structure
F1142-3225	CRIF1	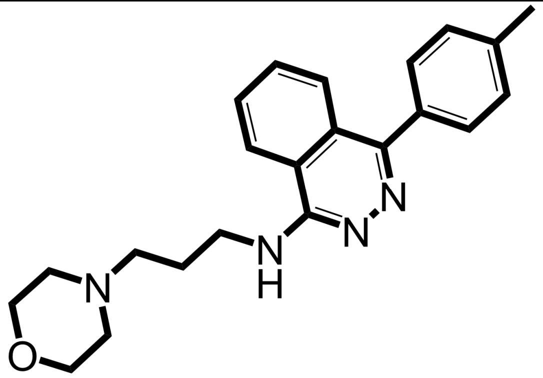
F0922-0913	CRIF1	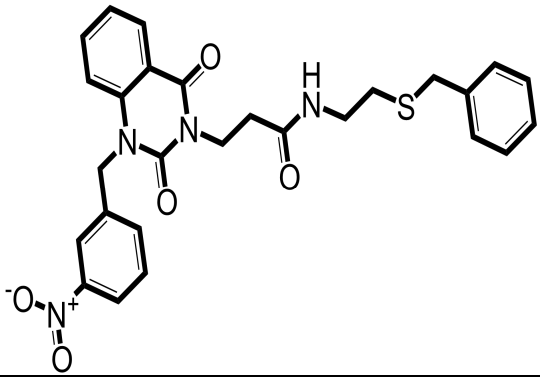
